# Computational identification of multiple lysine PTM sites by analyzing the instance hardness and feature importance

**DOI:** 10.1038/s41598-021-98458-y

**Published:** 2021-09-23

**Authors:** Sabit Ahmed, Afrida Rahman, Md. Al Mehedi Hasan, Shamim Ahmad, S. M. Shovan

**Affiliations:** 1 Computer Science and Engineering, Rajshahi University of Engineering and Technology, Rajshahi, 6204 Bangladesh; 2 Computer Science and Engineering, University of Rajshahi, Rajshahi, 6205 Bangladesh

**Keywords:** Functional genomics, Proteomic analysis, Post-translational modifications, Protein sequence analyses, Computational biology and bioinformatics, Computational models, Data processing, Machine learning, Protein function predictions, Proteome informatics, Statistical methods

## Abstract

Identification of post-translational modifications (PTM) is significant in the study of computational proteomics, cell biology, pathogenesis, and drug development due to its role in many bio-molecular mechanisms. Though there are several computational tools to identify individual PTMs, only three predictors have been established to predict multiple PTMs at the same lysine residue. Furthermore, detailed analysis and assessment on dataset balancing and the significance of different feature encoding techniques for a suitable multi-PTM prediction model are still lacking. This study introduces a computational method named ’iMul-kSite’ for predicting acetylation, crotonylation, methylation, succinylation, and glutarylation, from an unrecognized peptide sample with one, multiple, or no modifications. After successfully eliminating the redundant data samples from the majority class by analyzing the hardness of the sequence-coupling information, feature representation has been optimized by adopting the combination of ANOVA F-Test and incremental feature selection approach. The proposed predictor predicts multi-label PTM sites with 92.83% accuracy using the top 100 features. It has also achieved a 93.36% aiming rate and 96.23% coverage rate, which are much better than the existing state-of-the-art predictors on the validation test. This performance indicates that ’iMul-kSite’ can be used as a supportive tool for further K-PTM study. For the convenience of the experimental scientists, ’iMul-kSite’ has been deployed as a user-friendly web-server at http://103.99.176.239/iMul-kSite.

## Introduction

Post-translational modifications (PTM) refers to the covalent addition of certain functional groups to a protein after the translation process^1^. These modifications have significant effects on cellular processes and proteomic research, including cellular signalling, subcellular localization, protein folding, protein degradation, and are also linked to a wide variety of diseases^2,3^. Therefore, identifying and comprehending PTM sites is crucial for scientific investigations in disease identification, prevention, and drug developments^4,5^.

There are 20 amino acid residues, such as alanine (A), cysteine (C), lysine (K), arginine (R), etc. Modifications that occur at lysine (K) are named lysine modification or K-PTM. Single or multiple lysine residues may be modified individually or simultaneously where one residue can influence others. In other words, these covalent modifications can aid different K-PTM types, including acetylation, crotonylation, ubiquitination, methylation, butyrylation, succinylation, biotinylation, and ubiquitin-like modifications^6–8^. Though there are several computational tools for predicting various K-PTMs separately, to the best of the authors’ knowledge, only three multi-label prediction systems have been developed so far that can take care of the multiplex Lys residues^8–14^. Qiu et al. proposed iPTM-mLys in 2016^5^, which could predict four different types of modifications (i.e. acetylation, crotonylation, methylation, and succinylation) simultaneously. The vectorized sequence-coupling model with the random forest algorithm was applied to construct iPTM-mLys^5,15–17^. Hasan and Ahmad proposed mLysPTMpred in 2018^18^, where the dataset of iPTM-mLys was utilized to extract the sequence-coupled features, and the cost-sensitive SVM was used as a learning algorithm. The most recent multi-PTM prediction system proposed by Sua et al.^19^ has utilized the combination of sequence graph transform (SGT) and convolutional neural networks. All the multi-label predictors, as mentioned earlier, need significant improvement in terms of prediction quality. Furthermore, the number of simultaneous K-PTM prediction capabilities needs to be enhanced. Though there are a few dedicated predictors with multi-PTM prediction capability, all these proposed systems have been trained on the same dataset. Some challenges in this research area include constructing and preprocessing multi-label datasets from raw proteins, lacking multi-label proteins, handling data imbalance, reducing feature dimensions, developing multi-label classifications systems, using proper multi-label evaluation metrics etc. Therefore, adding more types of K-PTMs increases the complexity of this type of research. That might be the reason behind the existence of such a small number of multi-label predictors as well as only one benchmark dataset. Therefore, we have aimed to address these aforestated challenges and construct a highly efficient tool to meet the current demand in the study of post-translational modifications.

**Figure 1:**
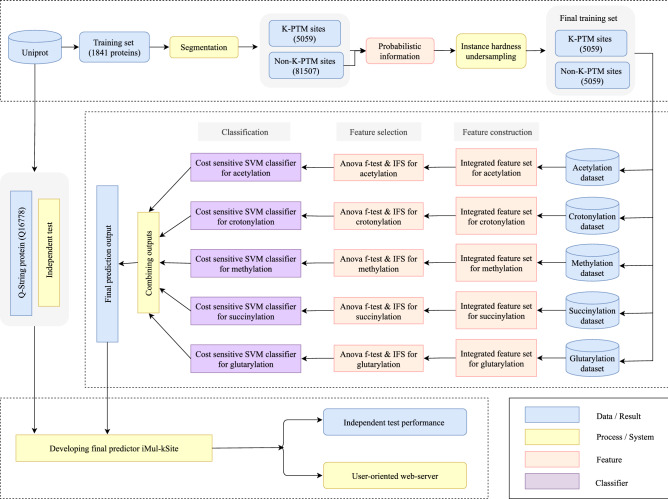
The system flowchart of iMul-kSite.

In this study, we have proposed a novel multi-label prediction system ’iMul-kSite’ to predict five different types of modifications (i.e. acetylation, crotonylation, methylation, succinylation, and glutarylation) concurrently. To develop a successful predictor for PTM sites, one of the main challenges is handling the imbalance in a dataset. Hence, the instance hardness (IH) based undersampling technique has been adopted to remove the redundant samples from the majority class. Another challenge is to elicit features from the input protein sequences as the appropriate features can play a crucial role in better prediction performance^18^. This study has considered several feature encoding methods to develop iMul-kSite, where the amino acid factors, encoded binary features^12,20^, pairs of k-spaced amino acids^21^, and the vectorized sequence-coupled model^5,10,15^ have been aggregated to encode a peptide segment. Afterwards, the analysis of variance (ANOVA) F test statistic along with the incremental feature selection approach has been used to eliminate the redundant and trivial features^22,23^. The support vector machine classifier with the variable cost adjustment process^18^ has been implemented to handle the imbalance in each benchmark dataset^24^. A 5-fold cross-validation^18^ scheme has been repeated five times for validating the statistical significance of the prediction results, and the average performance of each metric has been reported. A detailed overview is illustrated in Fig. 1.

## Methods

### Dataset construction

Accurate identification of protein’s post-translational modifications often requires a rigorously processed benchmark dataset. As this study is related to the multi-class multi-label classification problem, a few steps have been followed to construct five valid benchmark datasets.

#### Primary data collection and preprocessing

In the current study, human protein sequences have been utilized for prediction model development and benchmarking. About 9380 protein sequences have been collected from the Universal Protein Resource (UniProt)^25^ by applying various constraints (accessed 22 September 2020). Firstly, navigate to the ‘Advanced Search’ option, select the ‘PTM/Processing’ and ‘Modified residue [FT]’ option, keep ‘Any assertion method’ as ‘Evidence’. Then include another query space as ‘Organism [OS]’, choose ‘Homo sapiens (Human) [9606]’ from the suggestions as ‘Term’. Finally, select the ‘Reviewed’ option as the third field by adding one more query space. As this study is concerned with a multi-label classification problem, 5 different types of K-PTMs (i.e. acetylation, crotonylation, methylation, succinylation, and glutarylation) have been considered for the dataset construction. After applying a preliminary selection process with the specific keywords of each K-PTM, 1841 proteins have been obtained. For formulating peptide samples meticulously and comprehensively, Chou’s scheme^26^ has been adopted. According to this scheme, a peptide segment can generally be expressed by,

1 $$\begin{aligned} \small P_\zeta (K)=Q_{-\zeta } Q_{-({\zeta }-1)}...Q_{-2} Q_{-1} K Q_{+1} Q_{+2}...Q_{+({\zeta }-1)} Q_{+\zeta } \end{aligned}$$

where the symbol *K* denotes the responsible residue ’lysine’ at the centre, the subscript $\zeta$ being an integer, $Q_{-\zeta }$ and $Q_{+\zeta }$ denotes the $\zeta$th leftward and $\zeta$th rightward amino acid residues from the centre, and so forth. In this study, primarily a peptide sequence $P_\zeta (K)$ can be categorized into two types,

2 $$\begin{aligned} \small P_\zeta (K)\in {\left\{ \begin{array}{ll} P_\zeta ^+ (K),\text {if its center is K-PTM site} \\ P_\zeta ^- (K),\text {if its center is Non-K-PTM site} \end{array}\right. } \end{aligned}$$

where $P_\zeta ^+(K)$ contains the positive subset of the peptides and $P_\zeta ^-(K)$ contains the negative subset of the peptides with a lysine (*K*) residue at its centre, and the symbol $\in$ indicated the set theory relationship. For equal-sized K-PTM site formation, $(2\zeta +1)$-tuple peptide window with *K* at its centre has been employed. During segmentation, the lacking amino acid at both the right and left end has been filled with the nearest residue^5^. After the peptide fragments have gone through some screening, such as the elimination of sequences in case of redundancy, the primary dataset has been constructed with the following form,

3 $$\begin{aligned} \small S_\zeta (\text {K}) = S_\zeta ^+ (\text {K}) \cup S_\zeta ^- (\text {K}) \end{aligned}$$

where the positive subset $S_\zeta ^+ (\text {K})$ can contain any peptide samples which have one or more modifications (i.e. acetylation, crotonylation, methylation, succinylation, glutarylation) with *K* at the centre, while the negative subset $S_\zeta ^- (\text {K})$ can contain only the false K-PTM samples which have no modifications at all. The sliding window method^10^ was adopted to segment the protein sequences with different window sizes where $\zeta = 1, 2, 3, \ldots 24$. Based on the Accuracy value, window size was selected as $(2\zeta +1)= 49$ where $\zeta = 24$ (i.e. 24 right stream and 24 left stream amino acid residues). It should be mentioned that only the window sizes less than 51 were taken under consideration due to the compelling protein sequence length^10^. Therefore, Eq. (1) has been reduced to,

4 $$\begin{aligned} \small P(K)=Q_{-24} Q_{-23}...Q_{-2} Q_{-1} K Q_{+1} Q_{+2}...Q_{+23} Q_{+24} \end{aligned}$$

Following the aforestated process, 5059 K-PTM samples and 81507 Non-K-PTM samples have been obtained.

#### Data imbalance management and benchmark dataset formation

It can be observed that the primary dataset is highly imbalanced where the ratio between K-PTM and Non-K-PTM sites is  1:16. The instance hardness (IH) based undersampling technique has been employed for reducing this skewness^27^. Later at the classification level, a cost-sensitive SVM classifier has been utilized to address the imbalance in each K-PTM dataset.

##### Instance hardness undersampling

Smith, Martinez, and Giraud-Carrier have proposed the instance hardness (IH) undersampling technique for binary classification problems^27,28^. In this study, we adopted this technique by measuring the hardness of the sequence-coupling information which have been extracted from the primary dataset by using Eqs. (10), (11) and (12). The detailed methodology of the vectorized sequence-coupling feature extraction technique has been discussed in the “Feature construction” section. From Fig. 1, it can be observed that one or more modifications can occur at 5059 ’K-PTM’ samples, where 81507 ’Non-K-PTM’ samples lack any of the modifications. The objective here is to find out the most suitable peptide samples which represent no modification at all. In this work, the hardness of an instance in the coupling feature set measures how likely it is to be misclassified. Higher hardness values indicate that the data samples are noisy or on the border between ’K-PTM’ and ’Non-K-PTM’ classes, as the learning algorithms would cause them to overfit correctly^28^. For a peptide sample $(x_i, y_i)$, $p(y_i|x_i, h)$ denotes the conditional probability of label $y_i$ for the input feature vector $x_i$ given by the learning algorithms *h*. The higher the value of $p(y_i|x_i,h)$ is, the more likely *h* assigns the correct label to $x_i$, and it is quite opposite for the smaller value of $p(y_i|x_i,h)$^27,28^. The hardness of an instance $(x_i, y_i)$, concerning *h*, is defined as,

5 $$\begin{aligned} I_h[(x_i,y_i)]=1-p(y_i|x_i,h) \end{aligned}$$

Let ${\mathscr {H}}$ be the set of weak learners and *p*(*h*|*t*) be the corresponding weight of $h \in H$, where $t = {(x_i,y_i) : x_i \in X \wedge y_i \in Y }$. Hence, the hardness of an instance in the data sample takes the following form,

6 $$\begin{aligned} \begin{aligned} I[(x_i,y_i)]&=\sum _{\mathscr {H}}(1-p(y_i|x_i,h))p(h|t) \\&=\sum _{\mathscr {H}} p(h|t)-\sum _{\mathscr {H}} p(y_i|x_i,h)p(h|t) \\&= 1-\sum _{\mathscr {H}} p(y_i|x_i,h)p(h|t) \end{aligned} \end{aligned}$$

Following this concept, the imbalanced dataset has been resampled by eliminating the data points from the majority class with high instance hardness values, until the desired balancing ratio of 1:1 has been reached. To estimate the hardness of an instance, we utilized the cost-sensitive support vector machine^29–31^ which will be discussed later in this study. It should be mentioned that scikit-learn’s library^32^ has been used to implement the instance hardness (IH) based undersampling technique. Finally, 5059 positive and 5059 negative samples have been obtained, and the original peptide sequences with the expression of Eqs. (3) and (4) have been retrieved from the returned indices of the resampled dataset. The final benchmark datasets have been constructed by mapping the samples labeled as ’K-PTM’ and ’Non-K-PTM’ into each individual classes which takes the following form,

7 $$\begin{aligned} \small {\left\{ \begin{array}{ll} S_\zeta (\text {acetylation}) = S_\zeta ^+ (\text {acetylation}) \cup S_\zeta ^- (\text {acetylation}) \\ S_\zeta (\text {crotonylation}) = S_\zeta ^+ (\text {crotonylation}) \cup S_\zeta ^- (\text {crotonylation}) \\ S_\zeta (\text {methylation}) = S_\zeta ^+ (\text {methylation}) \cup S_\zeta ^- (\text {methylation}) \\ S_\zeta (\text {succinylation}) = S_\zeta ^+ (\text {succinylation}) \cup S_\zeta ^- (\text {succinylation}) \\ S_\zeta (\text {glutarylation}) = S_\zeta ^+ (\text {glutarylation}) \cup S_\zeta ^- (\text {glutarylation}) \\ \end{array}\right. } \end{aligned}$$

A comprehensive summary of dataset preparation has been presented in Fig. 1. The numbers of samples in the benchmark datasets are outlined in Table 1, and their detailed sequences and positions in the proteins are given in the Supplementary File. The distributions of different types of modifications in the benchmark datasets are tabulated in Table 2. It could be observed that our benchmark datasets contain 4089 samples belonging to one type of K-PTM, 861 to two types, 77 to three types, 26 to four types, and 6 to five types modifications.

**Table 1:** Number of samples in the benchmark dataset for different K-PTMs.

Attribute	$S_\zeta (acetylation)$	$S_\zeta (crotonylation)$	$S_\zeta (methylation)$	$S_\zeta (succinylation)$	$S_\zeta (glutarylation)$
True	4154	208	325	1253	236
False	5964	9910	9793	8865	9882

**Table 2:** K-PTM distributions in the training set.

Attribute	1 K-Type	2 K-Types	3 K-Types	4 K-Types	5 K-Types	Non-K-Types
Benchmark dataset	4089	861	77	26	6	5059

##### Cost-sensitive classifiers

We have handled the imbalance between the K-PTMs and Non-K-PTM sites by utilizing the instance hardness undersampling technique. However, it can be observed from Table  1 that still there exists some skewness between the positive and negative sites of each of the five modifications. Therefore, further adjustments are needed to deal with this issue. We have utilized five cost-sensitive SVM classifiers for mitigating the imbalance problem of five datasets in Table 1. A detailed discussion on the support vector machine prediction algorithm and the proposed model development are presented in the “Support vector machine” and “Model development and validation” sections respectively.

### Feature construction

With the evolution of the biological sequences, several encoding methods have been developed for extracting pertinent features hidden in the sequences. After preliminary analysis, it has been observed that the amino acid factors, encoded binary features, pairs of k-spaced amino acids, and the vectorized sequence coupling^12,15,20^ technique are more appropriate for representing the protein sequences of the multiple lysine modification sites than any other encoding methods.

#### Amino acid factors

Five multidimensional attributes^20,33^, which include polarity, secondary structure, molecular volume, electrostatic charge, and codon diversity^34^, have been constructed from AAIndex by using multivariate statistical analysis^12^. These five transformed properties can be introduced as amino acid factors (AAF)^34^. Since the AAF can reduce the dimensionality of the feature space of physicochemical properties efficiently, it has been utilized in many biological studies^12,34^. The dimensionality of feature vectors has been calculated as follows,

8 $$\begin{aligned} D= peptide\ sequence\ length \times number\ of\ factors \end{aligned}$$

With a peptide sequence of length 27 and previously described five amino acid factors, $49{\times }5 = 245$ dimension features have been derived by using this formula.

#### Binary encoding

Binary encoding^12^ can represent the amino acid position and composition by using 20 binary bits for one amino acid^12^. But one additional bit has been conjoined to handle the complexity of sliding windows. For 21 amino acids structured as ‘ACDEFGHIKLMNPQRSTVWYZ’, each residue inside a sequence fragment can be formed by a 21-dimension binary vector^12^. For instance, residue ‘*A*’, ‘*G*’ and ‘*Z*’ have been encoded as ‘100000000000000000000’, ‘000000100000000000000’ and ‘000000000000000000001’ respectively. According to this concept, each resultant peptide segment is expressed as $49{\times }21 = 1029$-dimensional feature vectors.

#### Pairs of k-spaced amino acids

The formation of k-spaced amino acid pairs encoding technique^12,21,35^ calculates the occurrence frequencies of the pairs of k-spaced amino acids from a segmented protein sample, that can express the short linear motif information out of it^12,30^. For instance, the encoding of a peptide segment will be a 441-dimensional feature vector if $k=0$. This can be defined as,

9 $$\begin{aligned} (N_{AnA}/N_{Total}, N_{AnC}/N_{Total},..., N_{YnY}/N_{Total})_{441} \end{aligned}$$

where *n* stands for any of amino acid, $N_{Total}$ means the occurrence frequency of all k-spaced amino acid pairs^35^ and $N_{AnA}$ means the occurrence frequency of the *AnA* pairs in the segment^20^ when $k=0$. In this study, after merging each of the 441-dimension feature vectors for $k = 0, 1, 2, 3, 4$, a total of 2205-dimensional features have been formed.

#### Sequence coupling

The composition of pseudo amino acid or PseAAC^10,36,37^ has been designed to preserve the sequence pattern information, which is a much harder task for any existing machine learning algorithm^38^. In this study, incorporating sequence coupling information into Chou’s general PseAAC has been adopted for extracting features from peptide sequences^5,15,18^. It can be defined as,

10 $$\begin{aligned} \small P(K) = P^+(K) - P^-(K) \end{aligned}$$

where,

11 $$\begin{aligned}&{P^+(K)=\Bigg [ P_{-24}^{C^{+}} P_{-23}^{C^{+}} \ldots P_{-1}^+ P_{+1}^+ \ldots P_{+23}^{C^{+}} P_{+24}^{C^{+}} \Bigg ]^T} \qquad \end{aligned}$$

12 $$\begin{aligned}&{P^-(K)=\Bigg [ P_{-24}^{C^{-}} P_{-23}^{C^{-}} \ldots P_{-1}^- P_{+1}^- \ldots P_{+23}^{C^{-}} P_{+24}^{C^{-}} \Bigg ]^T} \qquad \end{aligned}$$

where $P_{-24}^{C^{+}}$ in Eq. (11) denotes the conditional probability of amino acid $Q_{-24}$ at the leftmost position given that its adjacent right member is $Q_{-23}$ and so forth^5,18^. In contrast, only $P_{-1}^+$ and $P_{+1}^+$ are of non-contingent probability as *K* is the adjoining member of both amino acids at position $Q_{-1}$ and $Q_{+1}$. All the conditional probability values have been extracted from the positive training dataset. Additionally, all the probability values in Eq. (12) are identical to those of Eq. (11) other than that they can be derived from the negative training dataset. Thus, after omitting *K* from the center, $(49-1)=48$ dimension features have been obtained.

#### Feature ensembling

Initially, the four aforestated feature encoding techniques (i.e. AAF, BE, CKSAAP, and sequence coupling) have been implemented separately to encode the training peptides. However, for extracting more PTM-contextual information from the protein sequences, encoded features have been ensembled serially, and scaled through standardization. Finally, $(49{\times }5) + (49{\times }21) + (441{\times }5) + 48 = 3527$ dimension features have been obtained.

### Feature selection

Since the dimension of the encoded features is higher, irrelevant, and redundant features should be removed to avoid learning complexity. For this reason, the analysis of variance (ANOVA) F test statistic technique^22,39^ has been adopted. It tests the null hypothesis (i.e. all the means of different groups were equal) against the alternative hypothesis (i.e. all the means differed from each other). The one-way ANOVA can be defined as,

13 $$\begin{aligned} F= \frac{(n-k)\sum n_i(\overline{\mathrm{Y}}_{i.}-\overline{\mathrm{Y}}_{..})^2}{(k-1)\sum (n_i-1)s_i^2} \end{aligned}$$

where $$n=\sum _{i=1}^k n_i$$, $$\overline{\mathrm{Y}}_{i.}=Y_{i.}/{n_i}$$, $${\overline{Y}}_{..}=Y_{..}/{n}$$

and

$$s_i^2=\sum _{j=1}^{n_i} (Y_{ij}-{\overline{Y}}_{i.})^2/{({n_i-1})}$$.

It should be mentioned that the dot in $Y_{i.}$ indicates an aggregation over the j index^39^. Where $Y_{i.}=\sum _{j=1}^{n_i} Y_{ij}$ and $Y_{..}=\sum _{i=1}^{k}\sum _{j=1}^{n_i} Y_{ij}$. The calculated *F* values are used to rank the features. The discriminative capability of a predictor is better for higher *F* values.

### Support vector machine

The support vector machine (SVM)^29–31^, one of the dominant statistical learning algorithms was adopted as a core prediction algorithm. It seeks the optimum hyperplane with the highest margin between two groups^18,40^. Furthermore, it solves the problem of constraint optimization as described below

14 $$\begin{aligned} maximize_\alpha \sum _{i=1}^{n}\alpha _i - \frac{1}{2}\sum _{i=1}^{n}\sum _{j=1}^{n}\alpha _i\alpha _j y_i y_j k(x_i,x_j) \end{aligned}$$

Subject to: $\sum _{i=1}^{n}y_i\alpha _i=0,\quad 0\le \alpha _i\le C$, for all i$=1,2,3,...,n$. After involving the kernel function, the discriminant function of SVM took the following form

15 $$\begin{aligned} f(x)=\sum _{i}^{n}\alpha _i y_i k (x,x_i)+b \end{aligned}$$

In this paper, the radial basis function kernel^18,41^ was applied to construct SVM classifier and given by, $k(x_i,x_j)=exp(-{\gamma }\Vert x_i-x_j\Vert ^2)$, where $\gamma >0$^42^. As the benchmark dataset was highly imbalanced, different error cost (DEC)^18^ method had been used to tackle the class imbalance problem^24,43^. According to this approach, the SVM soft margin objective function was adjusted to allocate two costs for misclassification^12^, such as $C^+$ for the positive class instances and $C^-$ for the negative class instances

16 $$\begin{aligned} C^+ = C*W^+, \qquad C^- = C*W^- \end{aligned}$$

In Eq. (16), $W^+$ is the weight for the positive instances and $W^-$ is the weight for the negative instances and defined by

 $W^+ = \frac{M}{2*M_1},\quad W^- = \frac{M}{2*M_2}$ where *M* is the total number of elements, $M_1$ is the number of elements for the positive class, and $M_2$ is the number of elements for the negative class.

### Evaluation metrics

As shown in Table 1 and Supplementary Material, the total number of peptide samples are 10118 in total, of which 4154 are labelled with ‘acetylation’, 208 with ‘crotonylation’, 325 with ‘methylation’, 1253 with ‘succinylation’, 236 with ‘glutarylation’, and 5059 with ‘Non-K-PTM’. Since a sample can contain more than one labels, metrics for multi-label systems^5,18^ have been utilized instead of ordinary metrics for single-label systems^9,10,12,44^. According to Chou’s formulation^45^, the metrics for multi-label systems can be defined as,

17 $$\begin{aligned} {\left\{ \begin{array}{ll} Aiming = \frac{1}{N}\sum _{i=1}^{N}\left( \frac{\Vert Y_i \cap Y_i^\prime \Vert }{\Vert Y_i^\prime \Vert }\right) \\ Coverage = \frac{1}{N}\sum _{i=1}^{N}\left( \frac{\Vert Y_i \cap Y_i^\prime \Vert }{\Vert Y_i\Vert }\right) \\ Accuracy = \frac{1}{N}\sum _{i=1}^{N}\left( \frac{\Vert Y_i \cap Y_i^\prime \Vert }{\Vert Y_i \cup Y_i^\prime \Vert }\right) \\ Absolute-True = \frac{1}{N}\sum _{i=1}^{N}\left( \Delta {\Vert Y_i,Y_i^\prime \Vert }\right) \\ Absolute-False = \frac{1}{N}\sum _{i=1}^{N}\left( \frac{{\Vert Y_i \cup Y_i^\prime \Vert }-{\Vert Y_i \cap Y_i^\prime \Vert }}{L}\right) \end{array}\right. } \end{aligned}$$

where *N* and *L* are the total numbers of the samples and labels in the system respectively^5,18^, $\cup$ and $\cap$ denotes the ‘union’ and ‘intersection’ in the set theory, $||\,||$ means the operator acting on the set to calculate the number of its elements, $Y_i$ and $Y_i^\prime$ denotes the subset that contained all the labels experiment-observed and all the labels predicted for the $i^{th}$ sample respectively, and

$$\begin{aligned} \Delta (Y_i,Y_i^\prime )= {\left\{ \begin{array}{ll} 1, \text {if all labels in }Y_i^\prime \text { and }Y_i\text { are identical }\\ 0, \text {otherwise} \end{array}\right. } \end{aligned}$$

The metrics defined above have been applied effectively in several multi-label based systems^5,18^.

### Model development and validation

In this study, five separate SVM classifiers^18^ have been used to predict acetylation, crotonylation, methylation, succinylation, and glutarylation sites. Each of the classifiers has performed binary classification on the benchmark dataset described in Table 1. For all five K-PTM types, necessary features have been extracted by integrating multiple encoding methods and 100 optimal features with ANOVA F-test have been selected to train the models, as shown in Fig. 1. The radial basis function (RBF) kernel^40,46^ has been used for each SVM classifier. As there is a lack of details about the exact 5-way splits of the dataset^40^, five complete runs of 5-fold cross-validation have been executed^5,18,47^. The misclassification cost *C* has been calculated according to Eq. (16) for handling the data imbalance issue. In this study, libSVM’s default parameters (i.e. $C=1$ and $\gamma =1/$number of features) have been selected to train the model. Eventually, after training the five binary SVM classifiers with the appropriate hyperparameters, multi-label predictor iMul-kSite has been constructed by combining the outputs from these classifiers^40^, as depicted in Fig. 1. Five times repetition of the 5-fold cross-validation^40^ have produced five sets of values of all metrics, which are defined in the previous section. The average results of each multi-label metric have been taken to evaluate the final model. It should be mentioned that Matlab 2019a and python 3.7.3 have been utilized to implement the system.

## Results

### Incremental feature selection

The feature selection procedure has been implemented in two steps. Primarily, all the features have been tested with the analysis of variance (ANOVA) and the features with statistical significance have been obtained^48^. Hence, all of the 3527 features have been ranked according to the calculated *F* values.

**Figure 2:**
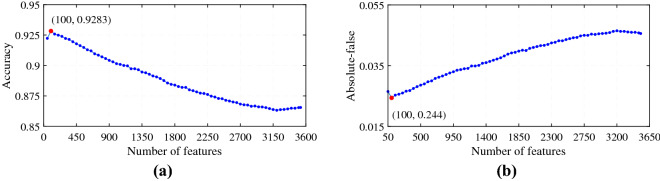
The IFS curves: (**a**) Feature range 50–3527 (Features vs. Accuracy). (**b**) Feature range 50–3527 (Features vs. Absolute-false).

Later, the incremental feature selection (IFS)^12^ algorithm has been applied for selecting the optimal number of features^12,48^. For each feature subset of top *m* ($m = 50, 100, 150, \dots , 3527$), one SVM classifier with libSVM’s default parameter^30,49^ has been trained for each K-PTM type and its accuracy and absolute-false rate have been measured by adopting 5-fold cross-validation. As depicted in Fig. 2, the highest accuracy of 92.83% with the lowest absolute-false rate of 2.44% has been achieved with 100 leading features. Finally, the proposed predictor kMul-iSite has been constructed by utilizing the top 100 features.

### Prediction performance of iMul-kSite

The performance of the iMul-kSite predictor derived from the aforementioned multi-label metrics is given in Table 3. The values of the five metrics are the average result of five times complete run of 5-fold cross-validation on the benchmark dataset. In Eq. (17), for the first four metrics, the higher the rate is, the better the performance will be, and for the last one, it is entirely the opposite^18^. The rate of the most crucial metric ‘Accuracy’ for our proposed predictor iMul-kSite is 92.83%. Besides, it has achieved a 93.36% ‘Aiming’ or ‘Precision’ rate which represents the average ratio of the predicted labels that hit the target of the original labels. The average ratio of the original labels that are covered by the hits of prediction referred to as ‘Coverage’ is 96.23%. To the best of the authors’ knowledge, no multi-label predictor has achieved a coverage rate of over 90% so far. In addition to that, the experimentally obtained rate of the most stringent and harsh metric ‘Absolute-True’ is 88.84% which is significant for any multi-label prediction system. Furthermore, the rate of ‘Absolute-False’ or ‘Hamming-Loss’ denoting the average ratio of completely wrong hits over the total prediction events is 2.44%.

### Comparison with existing multi-label predictors

According to the best of the authors’ knowledge, there are only three multi-label prediction systems that can predict multiple K-PTM sites simultaneously. All of these predictors have been constructed for identifying four types of K-PTMs i.e. acetylation, crotonylation, methylation, and succinylation. Qiu et al.^5^ have constructed iPTM-mLys, which is the first-ever multi-PTM prediction system for lysine modifications. Hasan and Ahmad^18^ have proposed another multi-label prediction system termed as mLysPTMpred. Recently, Sua et al.^19^ have constructed a method with the combination of convolutional neural network and sequence graph transform (CNN + SGT). The last two systems have achieved comparatively higher prediction performance than iPTM-mLys. They also have surpassed the milestone of reaching over 80% absolute-true rate.

**Table 3:** Cross-validation performance of the existing predictors.

Predictors	Functionality	Aiming(%)	Coverage(%)	Accuracy(%)	Absolute-True(%)	Absolute-False(%)
iPTM-mLys	4 K-PTMs	69.78	74.54	68.37	60.92	13.40
mLysPTMpred	4 K-PTMs	84.82	86.56	83.73	79.73	6.66
CNN + SGT$^{\mathrm{a}}$	4 K-PTMs	83.91	83.91	82.75	85.21	4.27
iMul-kSite $^{\mathrm{b}}$	4 K-PTMs*	$93.18 \pm (0.06)$	$96.13 \pm (0.09)$	$92.70 \pm (0.07)$	$88.77 \pm (0.08)$	$2.97 \pm (0.03)$
**iMul-kSite**$^{\mathrm{c}}$	**5 K-PTMs**	${\mathbf{93.36 }} \pm (0.05)$	${\mathbf{96.23 }} \pm (0.07)$	${\mathbf{92.83 }} \pm (0.07)$	${\mathbf{88.84 }} \pm (0.11)$	${\mathbf{2.44 }} \pm (0.02)$

The highest performance is indicated with bold texts.

$$^{\mathrm{a}}$$Method proposed by Nie et al.^19^$$^{\mathrm{b,c}}$$Correspond to the iMul-kSite performances on the benchmark datasets containing 4-PTMs and 5-PTMs respectively. *Corresponds to the 4 K-PTMs used in the previous studies i.e. acetylation, crotonylation, methylation and succinylation.

However, we have constructed a novel multi-PTM site predictor iMul-kSite which can predict 5 K-PTM sites concurrently. In addition to that, we have excluded the glutarylation sites from the benchmark dataset and reported the performance of iMul-kSite on the rest of the 4 K-PTMs in Table 3. In comparison with the recently developed multi-label predictor mLysPTMpred^18^, it can be observed that the rate of the most crucial metric ‘Accuracy’ for the proposed predictor iMul-kSite has been increased from 84.82% to 92.83%. Our proposed system has also achieved 8.54% and 9.67% increased aiming and coverage rates respectively. Furthermore, the absolute-true has reached 88.84% and the absolute-false has reached 2.44%. Therefore, the experimental results reported in Table 3 indicate that the constructed multi-label predictor iMul-kSite has achieved better performance than the existing state-of-art multi-PTM predictors even after the inclusion of one more type of PTM site prediction functionality^5,18,19^.

It should be mentioned that a Q-string protein sequence (Q16778) has been utilized in iPTM-mLys, mLysPTMpred, and Nie’s method for independent test^5,18,19^. Though these multi-PTM predictors do not account for glutarylation sites to be predicted, the independent test results of these predictors have been included in Table 4 for demonstrating the prediction accuracy of the proposed system. According to Eq. (17), the aiming, coverage, accuracy, and absolute-true rates are 95.00%, and the absolute-false rate is 1.67%. The superior performance obtained from both the cross-validation and independent test demonstrates the validity of our proposed model and it could be a high throughput tool for multi-label PTM site identification.

**Table 4:** Performance of different predictors on the Q-string independent test set.

Predictors	Functionality	Aiming (%)	Coverage (%)	Accuracy (%)	Absolute-true (%)	Absolute-false (%)
iPTM-mLys	4 K-PTMs	67.50	65.00	62.50	55.00	15.00
mLysPTMpred	4 K-PTMs	88.33	87.50	85.83	80.00	6.00
CNN + SGT $^{\mathrm{a}}$	4 K-PTMs	65.00	65.00	65.00	85.00	5.00
**iMul-kSite**	**5 K-PTMs**	**95.00**	**95.00**	**95.00**	**95.00**	**1.67**

The best achievable performance has been indicated with bold texts.

$^{\mathrm{a}}$Method proposed by Sua et al.^19^.

### Predictive performance of different feature encoding schemes

**Figure 3:**
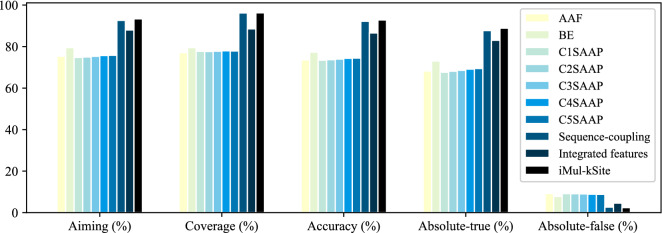
Performance comparison between different feature encoding techniques.

The performance obtained by iMul-kSite has been further compared with multiple baseline K-PTM prediction systems, developed using different feature extraction methods, such as the amino acid factors (AAF), binary encoding (BE), pairs of k-spaced amino acids (CKSAAP), and incorporation of sequence coupling information into general PseAAC^12,15,20,34,50,51^ to estimate iMul-kSite’s K-PTM related information extraction capability. The performances of the specified feature encoding schemes evaluated by 5-fold cross-validation are depicted in Fig. 3.

It may be observed that the amino acid factor (AAF) has acquired a higher absolute-false rate of 9.21% with considerably lower accuracy, absolute-true, aiming, and coverage rate. However, much better results have been picked up by binary encoding (BE) schemes. It has reached 77.35% accuracy with a 79.49% aiming rate and a 79.50% coverage rate. The absolute-false rate is reduced to 7.82% with an absolute-true rate of 73.05%. The composition of the k-spaced amino acid pairs (CKSAAP)^12,52^ encoding technique has been adopted for the different combinations of *k*, in which the ’0-spaced ($k = 0$) amino acid pairs’ has produced the lowest accuracy, aiming, coverage and absolute-true rate and the highest absolute-false rate. The performances secured by the composition of 1-spaced ($k = 0, 1$), 2-spaced ($k = 0, 1, 2$), and 3-spaced ($k = 0, 1, 2, 3$) amino acid pairs have been improved a little and maximized for the composition of 4-spaced ($k = 0, 1, 2, 3, 4$) amino acid pairs as illustrated in Fig. 3. It has achieved 74.44% accuracy, which is the topmost accuracy among the various combinations of CKSAAP encoding schemes but compared to other feature extraction techniques, it is not a desirable performance. Sequence-coupling, which is one of the most crucial encoding strategies, has attained a higher accuracy rate of 92.20%, an aiming rate of 92.62% with a much lower absolute-false rate of 2.66%. It has obtained a coverage rate above 90%, which is a rare example in bioinformatics. Therefore, integrating all the feature extraction methods has been considered a successful approach for developing a multi-label predictor. Consequently, the sequence-coupling has been combined with amino acid factor, binary encoding, and the composition of k-spaced amino acid pairs where *k* = 0, 1, 2, 3, 4. But the performances of the integrated features have been degraded and for 3527 dimension features, accuracy has been reduced to 86.55% with the increased absolute-false rate of 4.57%. Later, 100 optimal features have been selected from the high dimension features by conducting ANOVA F-test. By using the libSVM’s default parameter value of C and gamma, accuracy and aiming rate have been reached 92.83% and 93.36% respectively^49^. The most uncompromising metric absolute-true rate is 88.84% with a lower absolute-false rate of 2.44%. Figure 3 points out that the model constructed with the informative features termed as ’iMul-kSite’ has achieved a discernible performance among all the feature encoding techniques described earlier.

### Optimal features analysis

**Figure 4:**
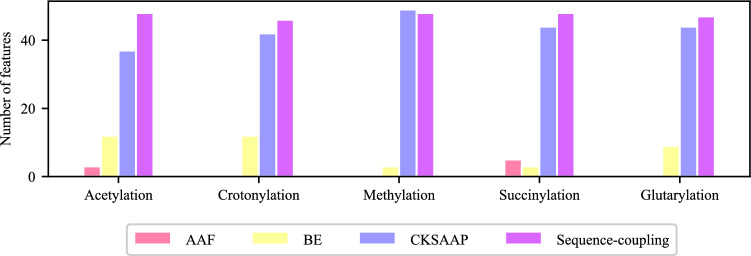
Feature distribution in the optimal feature sets.

The feature distribution for different K-PTM types is shown in Fig. 4. Moreover, the percentages of each type of feature selected with ANOVA and IFS are illustrated in Table 5 for a better understanding of the importance and dominance of the corresponding features. For the acetylation feature set, out of 100 optimal features, 3 belong to the AAF, 12 belong to the BE, 37 belong to the CKSAAP, and 48 belong to the sequence-coupling. Therefore, the ratios of selected dimensions of these four types of features are 1.23% (3/245), 1.17% (12/1029), 1.68% (37/2205), and 100% (48/48) respectively.

The crotonylation feature set comprises 46 sequence-coupling features, 12 BE features, and 42 CKSAAP features. Figure 4 and Table 5 show that the optimal feature set of crotonylation does not contain any of the AAF features. Hence, the selected dimension ratios of BE, CKSAAP, and sequence-coupling features are 1.17% (12/1029), 1.91% 42/2205), and 95.83% (46/48) respectively. Besides, the methylation feature set consists of 3 BE features, 49 CKSAAP features, and 48 sequence-coupling features, and the ratios of the selected dimensions for each type of feature are 0.29% (3/1029), 2.23% (49/2205), and 100% (48/48) respectively. For the succinylation dataset, 5, 3, 44, and 48 features belong to the AAF, BE, CKSAAP, and sequence-coupling respectively. The dimension ratios for AAF, BE, CKSAAP, and sequence-coupling are 2.04% (5/245), 0.29% (3/1029), 2.00% (44/2205), and 100% (48/48) respectively. For the glutarylation dataset, 9, 44, and 47 features belong to the BE, CKSAAP, and sequence-coupling respectively. The dimension ratios for BE, CKSAAP, and sequence-coupling are 0.88% (9/1029), 2.00% (44/2205), and 97.92% (47/48) respectively.

**Table 5:** Percentage of features selected with ANOVA F-Test and IFS.

Feature name	Acetylation	Crotonylation	Methylation	Succinylation	Glutarylation
AAF (%)	1.23	0.00	0.00	2.04	0.00
BE (%)	1.17	1.17	0.29	0.29	0.88
CKSAAP (%)	1.68	1.91	2.23	2.00	2.00
Sequence-coupling (%)	100.00	95.83	100.00	100.00	97.92

As reflected in Table 5, the selected feature dimensions for BE, AAF, and CKSAAP have varied over different types of K-PTM site prediction. The sequence-coupling features have a stronger influence on the identification of all of the five K-PTM sites. In contrast, BE, and CKSAAP features have much smaller and almost similar effects on each K-PTM site prediction. AAF features have a slightly better impact on the acetylation and succinylation site prediction but those have barely any effect on the crotonylation, methylation, and glutarylation site prediction. Therefore, it may be concluded that the proposed model augmented the sequence-coupling effect with the essential features of AAF, BE, and CKSAAP has intensified the prediction performance of iMul-kSite.

### Analysis on different modifications

The multi-label predictor iMul-kSite has been developed by combining outputs from the five optimized binary classifiers as discussed in the previous section. Though the final outputs have been evaluated by the multi-label metric system, each of the individual classifiers has been evaluated and tuned depending on the area under curve (AUC) value. From Table 1, it can be seen that the acetylation dataset is quite a balanced dataset. But The imbalance ratio of the number of succinylated sites to that of non-succinylated sites is approximately 1:7. On the other hand, the crotonylation, methylation and glutarylation datasets have higher imbalance ratios (around 1:40) between the number of positive and negative peptides. In this study, the imbalance between the positive and negative sites for different datasets has been handled in two stages. Firstly, the ‘K-PTM’ and ‘Non-K-PTM’ sites containing samples have been resampled at the dataset level. Later, the imbalance in each modification dataset has been minimized at the classifier level. It has been observed that, the average AUC of acetylation and succinylation classifiers were 97.64% and 98.44%, respectively. On the other hand, the average AUC values of crotonylation, methylation and glutarylation are 99.98%, 99.89% and 99.96% respectively. It can be concluded that after applying successful data balancing techniques at different levels, the constructed predictor iMul-kSite has demonstrated its superior performance for identifying all five types of different modifications.

### Web-server

To aid the experimental researches, a user-oriented web-server for iMul-kSite has been developed. It can be found at http://103.99.176.239/iMul-kSite where proper guidelines for submitting query protein sequences are provided. Users are allowed to submit query sequences either in the input box or in a batch file. For better understanding, a few protein sequences taken from the independent test dataset are included as examples. In addition to that, the benchmark dataset and the training features used for constructing iMul-kSite will be provided upon user request.

## Limitations

To improve the efficiency as well as to reduce the computational complexity of identifying 5 K-PTMs simultaneously, we considered instance hardness threshold (IHT) as an undersampling technique and incremental feature selection (IFS) with ANOVA F-Test as feature selection algorithm. Other structural features and evolutionary features might be utilized to improve the performance. Currently, our predictor iMul-kSite can deal with only five modifications i.e. acetylation, crotonylation, methylation, succinylation and glutarylation. We would include more types of modifications in our future study.

## Conclusion

Understanding the significance of identifying multiple lysine PTM sites, an efficient and successful predictor iMul-kSite has been developed with five lysine PTM sites prediction capability. After adopting successful data balancing methods, optimized features with the cost-sensitive learning algorithms have improved the prediction performance of the proposed predictor iMul-kSite significantly. Experimental outcomes demonstrate that iMul-kSite is highly promising compared to the existing state-of-the-art multiple lysine PTM site predictors. It is expected to become a high throughput tool for the experimental researchers for further PTM study on the lysine residues. Even experimental scientists may use this web-based tool without knowing its implementation details. Besides, a similar methodology of the proposed predictor can be used in the study of other PTMs such as C-PTM, R-PTM, and S-PTM that correspond to multi-label PTM sites at Cys, Arg, and Ser residues respectively. However, iMul-kSite was designed for five K-PTM types. To extend its prediction capability, other PTM types with new protein sequences can be added in the future.

## Supplementary Information

Supplementary Information.

## References

[1] Saraswathy N, Ramalingam P (2011). Concepts and Techniques in Genomics and Proteomics.

[2] McDowell, G. & Philpott, A. New insights into the role of ubiquitylation of proteins. In *International Review of Cell and Molecular Biology*, Vol. 325, 35–88 (Elsevier, 2016). 10.1016/bs.ircmb.2016.02.002 27241218

[3] Weissman, J. D., Raval, A. & Singer, D. S. Assay of an intrinsic acetyltransferase activity of the transcriptional coactivator CIITA. In *Methods in Enzymology*, Vol. 370, 378–386 (Elsevier, 2003). 10.1016/S0076-6879(03)70033-0 14712661

[4] Chou K-C (2015). Impacts of bioinformatics to medicinal chemistry. Med. Chem..

[5] Qiu W-R, Sun B-Q, Xiao X, Xu Z-C, Chou K-C (2016). iPTM-mLys: identifying multiple lysine PTM sites and their different types. Bioinformatics.

[6] Freiman RN, Tjian R (2003). Regulating the regulators: Lysine modifications make their mark. Cell.

[7] Xu Y, Chou K-C (2016). Recent progress in predicting posttranslational modification sites in proteins. Curr. Top. Med. Chem..

[8] Jia J, Liu Z, Xiao X, Liu B, Chou K-C (2016). iSuc-PseOpt: Identifying lysine succinylation sites in proteins by incorporating sequence-coupling effects into pseudo components and optimizing imbalanced training dataset. Anal. Biochem..

[9] Rahman, A., Ahmed, S., Rahman, J. & Hasan, M. A. M. Prediction of formylation sites by incorporating sequence coupling into general PseAAC. In *2020 IEEE Region 10 Symposium (TENSYMP)*, 921–924 (IEEE, 2020).

[10] Ahmed S (2021). predPhogly-Site: Predicting phosphoglycerylation sites by incorporating probabilistic sequence-coupling information into PseAAC and addressing data imbalance. PLoS ONE.

[11] Wu M, Yang Y, Wang H, Xu Y (2019). A deep learning method to more accurately recall known lysine acetylation sites. BMC Bioinform..

[12] Ju Z, He J-J (2018). Prediction of lysine glutarylation sites by maximum relevance minimum redundancy feature selection. Anal. Biochem..

[13] Bao, W., Yang, B. & Chen, B. 2-hydr\_ensemble: Lysine 2-hydroxyisobutyrylation identification with ensemble method. *Chemom. Intell. Lab. Syst.* 104351 (2021).

[14] Bao W (2019). Cmsenn: Computational modification sites with ensemble neural network. Chemom. Intell. Lab. Syst..

[15] Chou K-C (1993). A vectorized sequence-coupling model for predicting HIV protease cleavage sites in proteins. J. Biol. Chem..

[16] Chou K-C (1996). Prediction of human immunodeficiency virus protease cleavage sites in proteins. Anal. Biochem..

[17] Lin W-Z, Fang J-A, Xiao X, Chou K-C (2011). iDNA-Prot: Identification of DNA binding proteins using random forest with grey model. PLoS ONE.

[18] Hasan MAM, Ahmad S (2018). mLysPTMpred: Multiple lysine PTM site prediction using combination of SVM with resolving data imbalance issue. Nat. Sci..

[19] Sua JN (2020). Incorporating convolutional neural networks and sequence graph transform for identifying multilabel protein lysine PTM sites. Chemom. Intell. Lab. Syst..

[20] Zhe J, Wang S-Y (2020). Prediction of 2-hydroxyisobutyrylation sites by integrating multiple sequence features with ensemble support vector machine. Comput. Biol. Chem..

[21] Tung C-W (2013). Prediction of pupylation sites using the composition of k-spaced amino acid pairs. J. Theor. Biol..

[22] Chen D, Liu Z, Ma X, Hua D (2005). Selecting genes by test statistics. BioMed Res. Int..

[23] Ju Z, Wang S-Y (2019). iLys-Khib: Identify lysine 2-Hydroxyisobutyrylation sites using mRMR feature selection and fuzzy SVM algorithm. Chemom. Intell. Lab. Syst..

[24] Veropoulos K, Campbell C, Cristianini N (1999). Controlling the sensitivity of support vector machines. Proc. Int. Joint. Conf. AI.

[25] Consortium U (2019). UniProt: A worldwide hub of protein knowledge. Nucleic Acids Res..

[26] Chou K-C (2001). Prediction of signal peptides using scaled window. Peptides.

[27] Smith MR, Martinez T, Giraud-Carrier C (2014). An instance level analysis of data complexity. Mach. Learn..

[28] Le T (2018). A cluster-based boosting algorithm for bankruptcy prediction in a highly imbalanced dataset. Symmetry.

[29] Vapnik V (2013). The Nature of Statistical Learning Theory.

[30] Ju, Z. & Wang, S.-Y. Prediction of lysine formylation sites using the composition of k-spaced amino acid pairs via Chou’s 5-steps rule and general pseudo components. *Genomics* **112**, 859–866 (2020). 10.1016/j.ygeno.2019.05.027 31175975

[31] Cortes C, Vapnik V (1995). Support-vector networks. Mach. Learn..

[32] Pedregosa F (2011). Scikit-learn: Machine learning in Python. J. Mach. Learn. Res..

[33] Atchley WR, Zhao J, Fernandes AD, Drüke T (2005). Solving the protein sequence metric problem. Proc. Natl. Acad. Sci..

[34] Ju, Z. & He, J.-J. Prediction of lysine propionylation sites using biased SVM and incorporating four different sequence features into Chou’s PseAAC. *J. Mol. Gr. Model.* **76**, 356–363 (2017). 10.1016/j.jmgm.2017.07.022 28763688

[35] Ju Z, Cao J-Z (2017). Prediction of protein N-formylation using the composition of k-spaced amino acid pairs. Anal. Biochem..

[36] Chou K-C (2011). Some remarks on protein attribute prediction and pseudo amino acid composition. J. Theor. Biol..

[37] Du, P., Wang, X., Xu, C. & Gao, Y. PseAAC-Builder: A cross-platform stand-alone program for generating various special Chou’s pseudo-amino acid compositions. *Anal. Biochem.* **425**, 117–119 (2012). 10.1016/j.ab.2012.03.015 22459120

[38] Zhang Z (2011). Identification of lysine succinylation as a new post-translational modification. Nat. Chem. Biol..

[39] Kutner MH, Nachtsheim CJ, Neter J, Li W (2005). Applied Linear Statistical Models.

[40] Hasan MAM, Ahmad S, Molla MKI (2017). iMulti-HumPhos: A multi-label classifier for identifying human phosphorylated proteins using multiple kernel learning based support vector machines. Mol. BioSyst..

[41] Ruan, X., Zhou, D., Nie, R. & Guo, Y. Predictions of apoptosis proteins by integrating different features based on improving pseudo-position-specific scoring matrix. *BioMed Res. Int.* 2020 (2020). 10.1155/2020/4071508 PMC7201498 32420339

[42] Ma Y, Yu Z, Han G, Li J, Anh V (2018). Identification of pre-microRNAs by characterizing their sequence order evolution information and secondary structure graphs. BMC Bioinform..

[43] Batuwita, R. & Palade, V. Efficient resampling methods for training support vector machines with imbalanced datasets. In *The 2010 International Joint Conference on Neural Networks (IJCNN)*, 1–8 (IEEE, 2010).

[44] Chandra A, Sharma A, Dehzangi A, Shigemizu D, Tsunoda T (2019). Bigram-PGK: Phosphoglycerylation prediction using the technique of bigram probabilities of position specific scoring matrix. BMC Mol. Cell Biol..

[45] Chou K-C (2013). Some remarks on predicting multi-label attributes in molecular biosystems. Mol. Biosyst..

[46] Jiang, M. & Cao, J.-Z. Positive-Unlabeled learning for pupylation sites prediction. *BioMed Res. Int.* 2016 (2016). 10.1155/2016/4525786 PMC4992543 27579315

[47] Hasan MAM, Ahmad S, Molla MKI (2017). Protein subcellular localization prediction using multiple kernel learning based support vector machine. Mol. BioSyst..

[48] Semwal VB, Singha J, Sharma PK, Chauhan A, Behera B (2017). An optimized feature selection technique based on incremental feature analysis for bio-metric gait data classification. Multim. Tools Appl..

[49] Chang C-C, Lin C-J (2011). LIBSVM: A library for support vector machines. ACM Trans. Intell. Syst. Technol..

[50] Torkamani A, Schork NJ (2007). Accurate prediction of deleterious protein kinase polymorphisms. Bioinformatics.

[51] Ju Z, Wang S-Y (2020). Computational identification of lysine glutarylation sites using positive-unlabeled learning. Curr. Genomics.

[52] Chen Y-Z, Tang Y-R, Sheng Z-Y, Zhang Z (2008). Prediction of mucin-type O-glycosylation sites in mammalian proteins using the composition of k-spaced amino acid pairs. BMC Bioinform..

